# Impact of methylenetetrahydrofolate reductase C677T polymorphism on the efficacy of photodynamic therapy in patients with neovascular age-related macular degeneration

**DOI:** 10.1038/s41598-019-38919-7

**Published:** 2019-02-22

**Authors:** Francesco Parmeggiani, Carla Enrica Gallenga, Ciro Costagliola, Francesco Semeraro, Mario R. Romano, Roberto Dell’Omo, Andrea Russo, Katia De Nadai, Donato Gemmati, Sergio D’Angelo, Elena Bolletta, Francesco Saverio Sorrentino

**Affiliations:** 1 Department of Morphology, Surgery and Experimental Medicine, University of Ferrara, Ferrara, 44121 Italy; 2 Center for the Study of Inflammation of the University of Ferrara, Ferrara, 44121 Italy; 3 Department of Biomedical and Specialty Surgical Sciences, University of Ferrara, Ferrara, 44121 Italy; 4 Department of Medicine and Health Sciences, University of Molise, Campobasso, 86100 Italy; 5 Department of Medical and Surgical Specialties, Radiological Sciences, and Public Health, University of Brescia, Brescia, 25121 Italy; 6 Department of Biomedical Sciences, Humanitas University, Milan, 20090 Italy; 7 Center for Retinitis Pigmentosa of Veneto Region, Camposampiero Hospital, Azienda ULSS 6 Euganea, Padova, 35131 Italy; 8 Department of Surgical Sciences, Maggiore Hospital, Azienda USL Bologna, Bologna, 40124 Italy

## Abstract

The most severe visual impairments due to age-related macular degeneration (AMD) are frequently caused by the occurrence of choroidal neovascularization (CNV). Although photodynamic therapy with verteporfin (PDT-V) is currently a second-line treatment for neovascular AMD, it can be conveniently combined with drugs acting against vascular endothelial growth factor (anti-VEGF) to reduce the healthcare burden associated with the growing necessity of anti-VEGF intravitreal re-injection. Because the common 677 C > T polymorphism of the methylenetetrahydrofolate reductase gene (MTHFR-C677T; rs1801133) has been described as predictor of satisfactory short-term responsiveness of AMD-related CNV to PDT-V, we retrospectively examined the outcomes of 371 Caucasian patients treated with standardized, pro-re-nata, photodynamic regimen for 24 months. Responder (R) and non-responder (NR) patients were distinguished on the basis of the total number of scheduled PDT-V (TN-PDT-V) and change of best-corrected visual acuity (∆-BCVA). The risk for both TN-PDT-V and ∆-BCVA to pass from R to NR group was strongly correlated with CT and TT genotypes of MTHFR-C677T variant resulting, respectively, in odd ratios of 0.19 [95% CI, 0.12–0.32] and 0.09 [95% CI, 0.04–0.21] (P < 0.001), and odd ratios of 0.24 [95% CI, 0.15–0.39] and 0.03 [95% CI, 0.01–0.11] (P < 0.001). These pharmacogenetic findings indicate a rational basis to optimize the future clinical application of PDT-V during the combined treatments of AMD-related CNV, highlighting the role of thrombophilia to be aware of the efficacy profile of photodynamic therapy.

## Introduction

Age-related macular degeneration (AMD) [MIM 603075] is the most common cause of central blindness or low vision in the elderly population of the industrialized countries, i.e. in the areas defined by the World Health Organization as AMR-A (Amr, Region of the Americas), Eur-A (Eur, European Region), and WPR-A (WPR, Western Pacific Region)^1–3^. Caucasian populations are largely more affected by AMD than other ethnic groups^4–7^. There are two different clinical forms of sight-threatening AMD: (i) atrophic AMD (or dry AMD), characterized by atrophic changes of photoreceptors, retinal pigment epithelium (RPE), Bruch’s membrane, and choriocapillaris; and (ii) neovascular AMD (or wet/exudative AMD), complicated by choroidal neovascularization (CNV) and associated with RPE detachment, hemorrhages, exudates and/or cystoid macular edema^8–17^. At present, neovascular AMD is considered the most common cause of legal blindness in the Western countries^18–21^. In this fast-progressive form of AMD, CNV is caused by endothelial cell migration coming from choriocapillaris through weaker areas in Bruch’s membrane. The activation of several signaling pathways leads to a proliferating unit whose aberrant vessels, lacking of gap junctions, leak so that exudates and blood can spread through retinal layers^8,10,13,17^. Two therapeutic strategies are currently available to switch off AMD-related CNV and reduce its dramatic effects on patient’s vision: the intravitreally administrated drugs acting against vascular endothelial growth factor (anti-VEGF) and, as second-line treatment, the photodynamic therapy with verteporfin (PDT-V)^22–31^. In fact, during the last years, the care management of neovascular AMD is advanced from treatments able to minimize the vision loss or stabilize visual acuity (i.e. as-needed quarterly PDT-Vs)^22–26^, to those that allow a vision improvement (i.e. monthly, pro-re-nata or proactive regimes of intravitreal injections of anti-VEGF drugs)^27–31^. However, because of the critical drawback due to the growing burden of anti-VEGF drugs employment during the real-life clinical practice^32–34^, PDT-V has been combined with anti-VEGF in several different manners, allowing variable diminutions of anti-VEGF re-treatments^35–44^ but, in some protocols, also increasing the number of patients who experienced a minor visual gain than those treated with anti-VEGF alone^36–38^. Despite a huge debate on the need of improving the therapeutic management of neovascular AMD, the possible customization of PDT-V has not been fully explored to improve the decision-making process about this photothrombotic procedure targeting AMD-related CNV^32,33^. Therapeutic action of PDT-V is realized by a laser-light-induced shutdown of the neovascular complex that was previously photosensitized by the intravenous injection of verteporfin^45–50^. Even if demographic and clinical predictors, such as age of patient, baseline best-corrected visual acuity (BCVA) and baseline CNV dimension, have been evaluated to explain the remarkable heterogeneity of PDT-V outcomes in patients with neovascular AMD^51–53^, no unequivocal data were recorded also after the differentiation between different CNV subtypes which are traditionally categorized by fluorescein angiography (FA) in: (i) classic CNV (C-CNV), characterized by a well-demarcated hyperfluorescent area with an hypofluorescent margin in FA early phase and dye leakage obscuring the boundaries of the neovascular lesion during the late phase; (ii) predominantly classic CNV (PC-CNV), with the classic component occupying 50% or more of the entire neovascular lesion that includes occult CNV and all the fluorescence-blocking constituents; (iii) minimally classic CNV (MC-CNV), with the classic component occupying less than 50% of the neovascular complex; (iv) occult CNV (O-CNV), with no classic component^22–25^. In fact, within patients treated with PDT-V for AMD-related CNV, other factors appear to be implicated in those differences of therapeutic responsiveness, which are clearly noticeable reviewing the final outcomes of both randomized controlled trials^22–26^ and real-life clinical studies^54–56^. Focusing on a pharmacogenetic predictive approach, our previous investigations have pointed out the role of thrombophilic and anti-thrombophilic single nucleotide polymorphisms (SNPs) in modifying the result of PDT-V in Caucasian patients with neovascular macular degenerations^57–60^. In fact, several coagulation-balance SNPs are able to influence the response to PDT-V as a consequence of the multifaceted photo-thrombotic mechanism triggered by this therapeutic strategy within the neovascular complex^61–65^. Although the common 677 C > T polymorphism of the methylenetetrahydrofolate reductase gene (MTHFR-C677T; rs1801133) has been described as positive pro-thrombotic factor for the closing of C- and PC-CNVs secondary to AMD after a single PDT-V procedure^57^, its role in determining the long-term outcomes of photodynamic protocol has not yet been evaluated. The present retrospective study verified  whether, during a 2-year period of PDT-V regimen, the carriers of MTHFR-C677T polymorphism with AMD-related C- or PC-CNV required less photodynamic re-treatments than patients without this SNP, also comparing the final differences of BCVA change between these two study clusters.

## Results

The study cluster consisted of 371 eyes of 371 patients treated with standardized PDT-V protocol, according to the procedures of the Treatment of Age-Related Macular Degeneration with Photodynamic Therapy (TAP) study^22,23^, after the diagnosis of treatment-naïve AMD complicated by subfoveal C- or PC-CNV. In the course of the retrospectively selection of the study population, 98 patients were ruled out from the final statistical analyses owing to lack in follow-up visits (38 cases), not exact adhesion to the check timing (22 cases), not unequivocal interpretation of FA reading (14 cases), incomplete data collection (13 cases), or PDT-V complications such as RPE tear, massive retinal hemorrhage, and acute severe visual acuity decrease (11 cases). At baseline, the demographic and clinical attributes of these excluded patients were not statistically different with respect to those of the study cluster. In Table 1 are summarized the demographic and clinical characteristics of the analyzed cluster of patients, separated in responder (R) and non-responder (NR) to represent the percentage and mean values of the examined variables. In the final study population, patients with CC wild genotype of MTHFR-C677T polymorphism were 151, whereas 220 were carriers of polymorphic genotypes (CT in 176 cases and TT in 44 cases).

**Table 1:** Demographic and clinical characteristics of the study cluster separated in responders (R) and non-responders (NR) to photodynamic therapy with verteporfin (PTD-V) on the basis of the total number of PDT-V scheduled in each patient during the 24-month follow-up period (TN-PDT-V), and change of best-corrected visual acuity from baseline to final visit (∆-BCVA).

Study population (n = 371 patients with classic or predominantly classic AMD-related CNV)				
Baseline characteristics	PDT-V responder (TN-PDT-V from 1 to 4 PDT-V)	PDT-V non-responder (TN-PDT-V from 5 to 8 PDT-V)	PDT-V responder (∆-BCVA from −0.4 to 0.1 logMAR)	PDT-V non-responder (∆-BCVA from 0.2 to 0.7 logMAR)
No. of patients	174	197	206	165
Sex–Male/Female, n (%)	78 (44.8)/96 (55.2)	92 (46.7)/105 (53.3)	90 (43.7)/116 (56.3)	80 (48.5)/85 (51.5)
Mean age ± SD (range) – years	72.9 ± 7.2 (52–89)	74.1 ± 6.2 (58–92)	73.1 ± 7.2 (52–89)	74.1 ± 5.9 (58–92)
Mean BCVA ± SD (range) – logMAR	0.58 ± 0.22 (1.0–0.2)	0.60 ± 0.22 (1.0–0.2)	0.58 ± 0.22 (1.0–0.2)	0.60 ± 0.21 (1.0–0.2)
Mean CNV area ± SD (range) – micron^2^	2730 ± 1333 (697–5368)	2489 ± 1324 (638–5349)	2630 ± 1350 (697–5368)	2567 ± 1313 (638–5349)

AMD, age-related macular degeneration; CNV, choroidal neovascularization; SD, standard deviation; BCVA, best-correct visual acuity; logMAR, logarithm of the minimum angle of resolution.

The baseline comparisons between demographic and clinical characteristics of these CC, CT, and TT patients are shown in Table 2. No significant statistical differences were found among the three genotypic groups. No significant deviations from Hardy-Weinberg equilibrium and genotype distribution were observed comparing MTHFR-C677T polymorphism among a control group of healthy Caucasian individuals [n = 200; CC = 93 (46.5%); CT = 88 (44.0%); TT = 19 (9.5%)] and the study group [n = 371; CC = 151 (40.7%); CT = 176 (47.4%); TT = 44 (11.9%)].

**Table 2:** At-baseline comparisons between the demographic and clinical characteristics of patients with CC, CT, and TT genotypes of MTHFR-C677T polymorphism.

Study population (n = 371 patients with classic or predominantly classic AMD-related CNV)				
Baseline characteristics	MTHFR-677 CC genotype (n = 151)	MTHFR-677 CT genotype (n = 176)	MTHFR-677 TT genotype (n = 44)	P value
Sex–Male/Female, n (%)	69 (45.7)/82 (54.3)	78 (44.3)/98 (55.7)	23 (52.3)/21 (47.7)	NS*
Mean age ± SD (range) – years	73.4 ± 6.2 (55–88)	73.8 ± 6.8 (54–92)	73.4 ± 7.9 (52–89)	NS^†^
Mean BCVA ± SD (range) – logMAR	0.60 ± 0.21 (1.0–0.2)	0.58 ± 0.22 (1.0–0.2)	0.57 ± 0.25 (1.0–0.2)	NS^†^
Mean CNV area ± SD (range) – micron^2^	2633 ± 1335 (724–5368)	2602 ± 1336 (638–5341)	2498 ± 1328 (761–5131)	NS^†^

AMD, age-related macular degeneration; CNV, choroidal neovascularization; MTHFR, methylenetetrahydrofolate reductase; SD, standard deviation; BCVA, best-correct visual acuity; logMAR, logarithm of the minimum angle of resolution; *χ^2^ test; ^†^corrected t-test; NS, not significant.

In our cluster of patients with classic or predominantly classic AMD-related CNV, the mean number of PDT-V ± standard deviation (SD) administered through the 24-month follow-up period, including the baseline application and the subsequent as-needed treatments, was 4.6 ± 1.6 (range: from 1 to 8). This average of total PDT-V is rather lower in comparison with that observed at the end of the 24-month follow-up period in TAP trial, i.e. 5.6 treatments from the study onset^23^. After the standardized photodynamic protocol, among carriers of MTHFR-C677T wild CC genotype, the mean number of PDT-V ± SD was 5.4 ± 1.5 (range: from 1 to 8), whereas in patients with CT and TT genotypes the averages of PDT-V ± SD were, respectively, 4.1 ± 1.4 (range: from 1 to 8) and 3.5 ± 1.4 (range: from 1 to 7). The statistical evaluation of the influence of MTHFR-C677T polymorphism on the responsiveness to PDT-V in terms of total number of treatments scheduled in each patient during the 24-month follow-up period (TN-PDT-V) has been accomplished also considering patient’s age per 3-year increment, baseline BCVA and baseline CNV area. Multivariate logistic regression analysis showed that polymorphic CT and TT genotypes were strongly correlated with, respectively, a 5- and 10-time decreased risk for TN-PDT-V of passing from R to NR group, i.e. odds ratios (OR) equal to 0.19 [95% confidence intervals (CI), 0.12–0.32] and 0.09 [95% CI, 0.04–0.21] (P < 0.001). Furthermore, lower patient’s age (OR, 1.13 [95% CI, 1.02–1.26]; P < 0.02) and greater baseline CNV area (OR, 0.80 [95% CI, 0.68–0.96]; P < 0.01) performed as weak positive predictors for good responsiveness to PDT-V with regard to the extent of re-treatment necessity in the course of our as-needed therapeutic protocol (Table 3). Likewise, the same statistical analysis concerning the change of BCVA from baseline to final visit (∆-BCVA), pointed out that CT and TT genotypes were also associated with, respectively, a 4- and 30-time decreased risk for ∆-BCVA of passing from R to NR group, i.e. ORs equal to 0.24 [95% CI, 0.15–0.39] and 0.03 [95% CI, 0.01–0.11] (P < 0.001). No other putative predictors emerged as significant factors influencing the responsiveness to PDT-V with regard to the degree of satisfactory vision outcomes, i.e. recovery, stabilization or slight worsening of BCVA (Table 3). Finally, when ∆-BCVA was analyzed on a continuous scale, patients with a CC genotype worsened by 0.23 logMAR [95%CI, 0.20–0.26 logMAR], those with CT genotype worsened by 0.09 logMAR [95%CI, 0.06–0.12 logMAR; P < 0.001 vs. CC genotype] and patients with TT genotype remained stable [0.01 logMAR; 95%CI, 0.06-0.07 logMAR; P < 0.001 vs. CC genotype]. These estimates did not change after adjusting for age, BCVA and CNV area at baseline.

**Table 3:** Summary of the multivariate logistic regression analyses for the examined binary dependent variables, i.e. total number of photodynamic therapies with verteporfin (PTD-V) scheduled in each patient during the 24-month follow-up period (TN-PDT-V), and change of best-corrected visual acuity from baseline to final visit (∆-BCVA). PDT-V, photodynamic therapy with verteporfin; BCVA, best correct visual acuity; OR, odds ratio; CI, confidence intervals; CNV, choroidal neovascularization; MTHFR, methylenetetrahydrofolate reductase; NR, not relevant; NS, not significant.

Independent variables	Dependent variable TN-PDT-V		Dependent variable ∆-BCVA	
	P value	OR (95% CI)	P value	OR (95% CI)
Patient’s age per 3-year increment	0.02	1.13 (1.02–1.26)	NS	NR
Baseline BCVA	NS	NR	NS	NR
Baseline CNV area	0.01	0.80 (0.68–0.96)	NS	NR
MTHFR-C677T CT genotype	0.001	0.19 (0.12–0.32)	0.001	0.24 (0.15–0.39)
MTHFR-C677T TT genotype	0.001	0.09 (0.04–0.21)	0.001	0.03 (0.01–0.11)

## Discussion

The current study pointed out that the thrombophilic MTHFR-C677T polymorphism, already recognized as positive predictor in short-term angiographic response to PDT-V of AMD-related C- and PC-CNVs^57^, get better the long-term outcomes of standardized, pro-re-nata, PDT-V protocol among patients with neovascular AMD. As a matter of fact, after two years of follow-up, the carriers of CT or TT genotype of MTHFR-C677T variant experienced a minor reduction of final BCVA as compared to non-carriers, showing a very low risk to pass from R to NR patients. Similarly, these polymorphic MTHFR-C677T genotypes are predictive of a minor probability to receive a greater number of PDT-V to deactivate CNV thus having to be included within NR patients. In particular, the chance to pass from R- to NR-status related to CT heterozygosis was remarkably higher  than that  related to TT homozygosis, indicating the existence of strong gene-dosage effect where the possibility of good response to PDT-V appears to be proportional to the number of the polymorphic T-alleles. Our retrospective investigation failed to document that demographic or clinical factors were important predictors of PDT-V outcomes in patients with neovascular AMD^51–53^. In fact, only considering the amount of the photodynamic re-treatments but not in regards to the final BCVA change, patient’s age and baseline CNV dimension weakly influenced the responsiveness to PDT-V. Besides, within our clusters of Caucasian ethnicity, the difference in genotype distribution of MTHFR-C677T between healthy controls and patients with neovascular AMD was appreciable even if  not significant, in line with the data previously reported in Asians by Tanaka and coworkers^66^.

Genotypic predictors act as factors influencing PDT-V effect because they modify the photo-thrombotic and immuno-inflammatory interactions between PDT-V targets (i.e. CNV with its blood content and endothelial wall) and degenerated retina-RPE structures in which the neovascular complex develops^57–65,67–69^. Considering the photo-thrombotic action of PDT-V on CNV^45–49^, a comprehensive appraisal of the thrombophilic predisposition of vascular endothelium induced by MTHFR-C677T-related hyperhomocysteinemia (HHcy) provides explanation about the rationale of this pharmacogenetic correlation^62–64^. Experimental and clinical findings point out that CNV therapeutic occlusion after PDT-V is obtained by the laser-light-triggered thrombosis of photosensitized neovascular network by means of three synergistic mechanisms of action: (i) cellular, (ii) vascular, and (iii) immunological^45–49^. PDT-V efficacy is due to the preferential binding of a specific photosensitizer, i.e. verteporfin, to the endothelium of CNV in comparison with that covering the walls of normal retino-choroidal vasculature of macular area. Verteporfin couples with low-density lipoproteins (LDL) to form a complex that is prevalently up-taken into neovascular endothelial cells because of their over-expression of LDL receptors. Post-PDT-V changes of neovascular endothelium are caused by the photo-oxidative action of several reactive oxygen species (ROS), which act as triggers able to achieve the therapeutic hemostasis inside CNV. ROS-related exposure of vascular basement membrane activates adhesion, degranulation, and aggregation of the platelets, followed by the release of vasoactive mediators that amplify platelet activation, thrombosis, vasoconstriction, and increased vascular permeability. This series of events finally causes blood stasis, tissue hypoxia, and variable extent of CNV occlusion^45^, which is also related to iatrogenic damages of choriocapillaris because of non-selective hemodynamic drop of sub-retinal micro-vasculature^48^. During PDT-V, the photochemical changes triggered at the level of CNV endothelium can be strengthened by the MTHFR-677 T-allele^62–64^, as supported by our results of good therapeutic responsiveness among carriers of CT and TT genotypes characterized by an evident gene-dosage effect. MTHFR-C677T polymorphism is a very common folate-pathway genotypic variant heterogeneously distributed in the various ethnic groups^70–73^. The environmental selective pressure modifies this SNP distribution reliably because of different protein-intake habits: in fact, TT homozygosity is particularly represented in Mexico, South Italy and North China with an evident continental north/south gradient in Western Countries conversely to a south/north gradient in Eastern ones^70,74^. Pro-thrombotic consequences of the MTHFR-677 T-allele are mainly due to its HHcy-related effect. This polymorphic allele affects homocysteine (Hcy) metabolism elevating its plasma level and altering both vascular wall structure and blood coagulation system, to result in a structural dysfunction that alters normal homeostatic properties of endothelium, including its role in regulation of vascular tone, hemostasis, and inflammation^75–80^. In particular, the HHcy thrombotic diathesis is related to the tissue factor, a membrane glycoprotein generating the coagulation process through thrombin boost^62,81,82^. Genetic predisposition to HHcy can justify the variable efficacy of phototrombotic treatments, such as PDT-V. The mechanisms by which a photodynamic procedure elicits its therapeutic effects triggering CNV endothelium are basically overlapped with those causing pro-thrombotic phenomena due to hyperhomocysteinemic, folate-related, gene variants. These SNPs produce functional damages in MTHFR and also in other enzymes regulating the methionine-homocysteine metabolism, reducing its activity and inducing thrombophilia by means of endothelial cells and platelets hyper-activation^45,62,75–80^. HHcy causes oxidative and inflammatory changes in blood vessels as consequence of ROS-related stimulation, followed by lipid peroxidation in membranes of endothelial cells and in circulating LDL, over-expression of lectin-like oxidized LDL receptor-1 and aberrant platelet activation. The importance of these interactions among HHcy- and PDT-V-related effects are also corroborated by a rational interpretation of the pharmacogenetic correlation observed in the present study and in a previously published series of patients treated with PDT-V for neovascular AMD, both indicative of an intriguing gene-environment relationship between photodynamic action and MTHFR-C677T polymorphism^57,62,83–91^. This interaction has been described as one of main factor causative of acute vision loss associated with a  photodynamic procedure^92^ and, of course, it should be taken into account in case of unexpected, PDT-V-related, adverse events such as severe BCVA decrease possibly owing  to a non-selective hyper-thrombotic response of the normal macular vasculature^93–96^. On the other hand, also when patients were repeatedly treated with PDT-V, several cases of atrophic RPE changes have been reported in the irradiated areas with previously normal RPE, indicating an iatrogenic damage due to choriocapillaris hypo-perfusion with consequent RPE damage especially occurred during long-term PDT-V regimen^48,97–100^. Likewise, macular RPE atrophy have been also observed after anti-VEGF intravitreal administrations^101–103^ and, as reported by Abdelfattah and coworkers, its extent is proportionally correlated with the number of the injections^101^.

Although neovascular AMD is traditionally considered one of the leading causes of irreversible vision loss in the developed Countries, the large-scale utilization of anti-VEGF drugs, particularly evident in the last 10 years, allowed an outstanding reduction of AMD patients eligible for legal blindness certification^104–106^, preserving or restoring their vision-related quality of life^107–109^. However, in the real-life clinical practice, none of these benefits can be obtained without burdening the healthcare system that is weighted down by the continuous increase of the necessity for  both monitoring and retreating of the patients with neovascular AMD^110^. In order to balance costs with benefits, one of the most promising solution is the synergistic or additive use of anti-VEGF drugs and PDT-V^111^. At present, these are the therapeutic strategies approved for the care of AMD-related CNV representing, in the clinical practice, the only treatments that can be simultaneously utilized to minimize both the irreversible vision loss caused by neovascular AMD and the socio-sanitary burden of this sight threatening disease. However, anti-VEGF/PDT-V combination is also characterized by an increased risk of detrimental effect in patient’s visual acuity secondary to RPE atrophy in macular area^98–103^.

Clinical investigations comparing the anti-VEGF/PDT-V combination with the anti-VEGF monotherapy always showed a lower necessity of anti-VEGF re-injection in the combination study groups^35–44,112^, whereas the mean BCVA gain has been often higher in the groups of patients exclusively treated with anti-VEGF drugs in comparison with those undergoing combined approach^36–38,112^. An ideal therapeutic management of a pathologic condition requiring a long-term patient’s take-in-charge, such as AMD-related CNV, should be able to individually select the most efficient strategies and the most efficient regimen for each of them, to reduce the gap between the need of patient-centered care and the actual care’s feasibility inside the healthcare provider.

Although PDT-V is no longer used as first-line therapy for neovascular AMD, the appraisal of its role as adjunctive treatment to anti-VEGF drugs has not yet led to a harmonized decision-making process^32,33,110^, generating heterogeneous clinical data^35–44,112^ which could find meaning through a new personalized pharmacogenetic approach for PDT-V application that might be inclusive of fundus autofluorescence imaging to inspect RPE atrophy^98–103^ and optical coherence tomography angiography to decide on single or combined retreatment. Consequently, predictive data on CNV response to PDT-V mono-therapy can represent the bases on which rationally expand our knowledge to optimize the modalities of synergistic combination between anti-VEGF and PDT-V effects^32,50,111,113,114^. In a perspective of translational medicine, the present findings on the common MTHFR-C677T polymorphism should be considered: (i) for the assessment of microvascular thrombus formation and expansion-rate in patients treated with photodynamic therapy to reduce the growth of solid tumors^115–117^; and, together with other consolidated pharmacogenetic data on anti-VEGF agents^118,119^, (ii) for the revision of the multifaceted  responsiveness time-to-time reported in patients treated with anti-VEGF/PDT-V combinations for neovascular AMD.

## Methods

### Patients enrolled in the study

In the course of this multicenter study, the clinical records of Caucasian patients exclusively treated with PDT-V for the occurrence of newly diagnosed AMD-related CNV were retrospectively examined, exclusively including those homogeneous data which had been collected in six Eye Clinics from March 2004 to September 2017. All phenotypic and genotypic findings were computed and analyzed at the University of Ferrara (Italy). In the majority of these patients (268 of 371), the 24-month PDT-V protocol had been completed before anti-VEGF drugs (pegaptanib, ranibizumab, aflibercept, and bevacizumab) become available in the normal clinical practice. Conversely, in each of the other cases subsequently treated (103 of 371), contraindications to the intravitreal administration of anti-VEGF drugs, objective compliance’s troubles or patient’s refusal of this invasive therapy were present. All the selected patients had undergone both FA and indocyanine green angiography (ICGA) at baseline examination to finalize an accurate differential diagnosis between C-, PC-, MC- and O-CNV. The CNV classification was based on definitions from the Treatment of Age-Related Macular Degeneration with Photodynamic Therapy (TAP) study, the Visudyne in Photodynamic Therapy study, and the Visudyne in Minimally Classic Choroidal Neovascularization study^22–24^. For our investigative purposes focusing the MTHFR-C677T pharmacogenetic aspect of PDT-V-related photothrombosis, only treatment-naïve patients suffering from AMD complicated by C- or PC-CNV were computed together to achieve a homogeneous study cluster, because of the lack of any significant difference in PDT-V responsiveness between these two similar CNV patterns^22,23,25,57,62^. On the other hand, considering the lack of any plausible correlation between PDT-V responsiveness and MTHFR-C677T polymorphism in patients with MC- and O-CNV^58,62^, these latter CNV patterns have been excluded from our data revision. As well, also subjects who had no regularly and/or correctly completed PDT-V protocol for a 24-month follow-up period have been ruled out. In particular, we have analyzed only the data of patients in whom the standardized PDT-V protocol was uneventfully completed in accordance with TAP study procedures^22,23^, also excluding patients with serious PDT-V complications such as RPE tear, massive retinal hemorrhage, and acute severe visual acuity decrease. Re-treatments were scheduled according to the international guidelines for PDT-V application, which recommend a patient’s examination at 3-month intervals and an additional course of treatment in case of persistent angiographic signs of CNV activity; each photodynamic treatment was scheduled within one week after baseline or follow-up angiographic exams^22,23^. Inclusion criteria are summarized as follows: (i) diagnosis of AMD in Caucasian patients with more than 50 years; (ii) BCVA better than 20/200 Snellen equivalent; (iii) angiographic diagnosis of C- or PC-CNV secondary to AMD after the examination of both FA and ICGA; (iv) active CNV under the geometric center of the foveal avascular zone; (v) greatest linear dimension of entire neovascular complex less than 5400 microns. On the other hand, exclusion criteria are listed herein: (i) history of any other anti-CNV treatment before and/or during the 24-month PDT-V protocol; (ii) angiographic diagnosis of MC- or O-CNV secondary to AMD after the examination of both FA and ICGA; (iii) any other possible cause of CNV different from AMD, such as pathologic myopia, angioid streaks, chorioretinal inflammatory diseases, hereditary retinal disorders, presumed ocular histoplasmosis syndrome, and/or severe ocular trauma; (iv) ascertained or suspected diagnosis of retinal angiomatous proliferation or polypoidal choroidal vasculopathy; (v) intraocular surgery and any laser-treatment of the eye during the 6 months before or the 3 months after the 24-month PDT-V protocol; (vi) presence of any significant condition, side effect and/or event possibly influencing the outcome of each PDT-V. At baseline and follow-up visits, these patients had undergone complete clinical examination, including medical and ophthalmologic anamneses, auto-refraction, BCVA test, slit-lamp biomicroscopy of the anterior segment, applanation tonometry, 60-diopter lens ophthalmoscopy, FA and ICGA. BCVA was measured using a standard logarithmic chart at a test distance of three meters. BCVA values (Snellen equivalent) were converted to the logarithm of the minimum angle of resolution (logMAR) scale for the statistical analyses. After a detailed description of the aims and procedures, patients gave their written informed consent to participate. In each studied patient, blood sample was collected for genotyping from September 2015 to December 2017. Genomic DNA was isolated from peripheral blood using standard proteinase K treatment, followed by phenol-chloroform extraction and ethanol precipitation. In a Peltier Thermal Cycler apparatus, samples were polymerase chain reaction (PCR)-genotyped for MTHFR-C677T polymorphism according to our earlier report^120^. MTHFR-C677T genotypes were confirmed by re-genotyping a random selection of samples. No discrepancies were found between genotypes determined in duplicate. All examinations were carried out in a blinded fashion in respect to the clinical data of each patient. The study followed the tenets of the Declaration of Helsinki and was approved by the Institutional Review Board of the University of Ferrara (protocol identification code PRUA2-2013-00002008; subgroup analysis of the version V3 amended on 12 June 2015 and approved on 10 September 2015).

### Statistical analysis

Considering the typology of the investigated parameters, the sample size calculation, accomplished for the amount of the selected patients (371 cases), provided a value constantly upper than 85%. This test was performed using the PASS 97 statistical program (NCSS Inc., Kaysville, UT, USA). In the study population, the expected genotype distribution of MTHFR-C677T polymorphism was checked by Hardy-Weinberg equilibrium test, and compared with a cluster of normal individuals matched for sex, age and ethnicity with the study group. At baseline, demographic and clinical characteristics were compared between patients with CC, CT, and TT MTHFR-C677T genotypes. A t-test, corrected for groups, was employed to compare the mean of continuous measures, and the χ^2^ test was used to compare proportions of categorical measures. In the present study, the main outcome measures were: (i) the total number of PDT-V scheduled during the 24-month therapeutic protocol (TN-PDT-V); and (ii) change of BCVA from baseline to 24 months after the first PDT-V (∆-BCVA). For our analytical purposes regarding the influence of MTHFR-C677T polymorphism on PDT-V efficacy, these main outcome measures have been considered as dependent variables and were arbitrarily categorized in two clinical levels of therapeutic responsiveness distinguishing responder (R) and non-responder (NR) patients on the basis of both TN-PDT-V and final ∆-BCVA (Table 4). Considering the expected mean clinical outcomes obtainable by an as-needed quarterly, standardized PDT-V regimen in patients with subfoveal AMD-related C- or PC-CNV, a case has been labeled as responder if a maximum of four TN-PDT-V in 24 months were able to control the disease, because this is the first number lower than both TAP-study average of 5.6^23^ and our average of 4.6 treatments. On the other hand, owing to the fact that in these patients the maximum estimated mean benefit of PDT-V protocol on ∆-BCVA is a slight vision loss or a visual acuity stabilization^25,26^, a case has been labeled as responder if a maximum of 0.1 logMAR BCVA reduction occurred at the end of 2-year follow-up. Odds ratios (OR) and 95% confidence intervals (CI) for combined comparisons were calculated by binary logistic regression models. Linear regression was used to estimate differences in ∆-BCVA across genotype subgroups. Multivariate analyses were performed to determine which factors were predictive of different response to PDT-V, using R/NR TN-PDT-V and R/NR ∆-BCVA as binary dependent variables. In these regression models, putative predictors were included according to the clinical plausibility of their possible influence on the dependent variables, i.e. TN-PDT-V and ∆-BCVA. Therefore, the following parameters were collectively examined as PDT-V predictors: patient’s age per 3-year increment, baseline BCVA, baseline CNV area, CT and TT genotypes of MTHFR-C677T^51–53,57^. Statistical analyses were performed by SySTAT V.5.0 (SySTAT Inc., Evanston, IL, USA) and SPSS Statistical Package (SPSS Inc., Chicago, IL, USA). A probability of P 0.05 was considered significant. During the investigation, the clinicians, laboratory personnel, and statistician were completely masked to both therapeutic interventions and clinical/genotyping outcomes regarding each enrolled patient.

**Table 4:** Criteria to distinguish responders (R) and non-responders (NR) to photodynamic therapy with verteporfin (PTD-V) on the basis of the total number of PDT-V scheduled in each patient during the 24-month follow-up period (TN-PDT-V), and change of best-corrected visual acuity from baseline to final visit (∆-BCVA). PTD-V, photodynamic therapy with verteporfin; TN-PDT-V, total number of photodynamic therapies with verteporfin; BCVA, best-corrected visual acuity; ∆-BCVA, change of best-corrected visual acuity; logMAR, logarithm of the minimum angle of resolution.

	PDT-V responder (R)	PDT-V non-responder (NR)
TN-PDT-V	from 1 to 4	from 5 to 8
∆-BCVA – logMAR	from −0.4 to 0.1	from 0.2 to 0.7

## Data Availability

All authors agree to make materials, data and associated protocols promptly available to readers.
